# Supplementary figures and images for: Cancer disrupts sex hormone-inflammation relationships: Analysis of ALI in males from NHANES 2007–2018
Source: PLoS One. 2025 Jun 18;20(6):e0325796. doi: 10.1371/journal.pone.0325796 (PMC12176118; doi:10.1371/journal.pone.0325796)

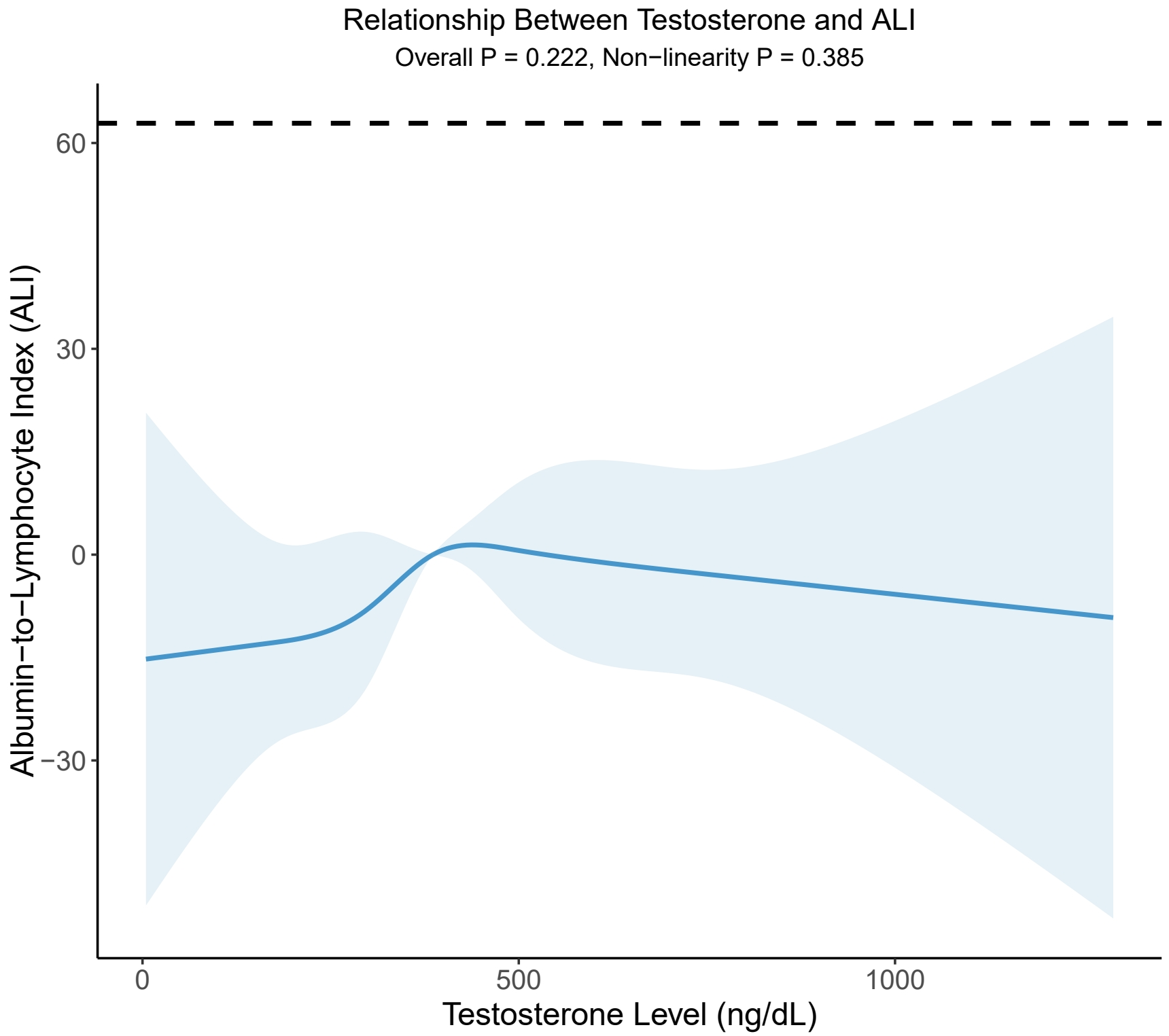

Supplement: S1 Fig — (TIF) [file pone.0325796.s001.tif]
